# Gut microbiota alternation with training periodization and physical fitness in Japanese elite athletes

**DOI:** 10.3389/fspor.2023.1219345

**Published:** 2023-07-14

**Authors:** Nobuhiko Akazawa, Mariko Nakamura, Nobuhiko Eda, Haruka Murakami, Takashi Nakagata, Hinako Nanri, Jonguk Park, Koji Hosomi, Kenji Mizuguchi, Jun Kunisawa, Motohiko Miyachi, Masako Hoshikawa

**Affiliations:** ^1^ Department of Sports Research, Japan Institute of Sports Sciences, Tokyo, Japan; ^2^ Faculty of Sport Sciences, Waseda University, Saitama, Japan; ^3^ Department of Sports Sciences, Japan Institute of Sports Sciences, Tokyo, Japan; ^4^ Department of Fundamental Education, Dokkyo Medical University, Tochigi, Japan; ^5^ Department of Physical Activity Research, National Institutes of Biomedical Innovation, Health and Nutrition, Tokyo, Japan; ^6^ Laboratory of Gut Microbiome for Health, Microbial Research Center for Health and Medicine, National Institutes of Biomedical Innovation, Health and Nutrition, Osaka, Japan; ^7^ Artificial Intelligence Center for Health and Biomedical Research, National Institutes of Biomedical Innovation, Health and Nutrition, Osaka, Japan; ^8^ Laboratory of Vaccine Materials and Laboratory of Gut Environmental System, Microbial Research Center for Health and Medicine, National Institutes of Biomedical Innovation, Health and Nutrition, Osaka, Japan; ^9^ Institute for Protein Research, Osaka University, Osaka, Japan

**Keywords:** gut microbiota, stool condition, fitness, training season, athlete

## Abstract

**Introduction:**

The gut microbiome plays a fundamental role in host homeostasis through regulating immune functions, enzyme activity, and hormone secretion. Exercise is associated with changes in gut microbiome composition and function. However, few studies have investigated the gut microbiome during training periodization. The present study aimed to investigate the relationship between training periodization and the gut microbiome in elite athletes.

**Methods:**

In total, 84 elite athletes participated in the cross-sectional study; and gut microbiome was determined during their transition or preparation season period. Further, 10 short-track speed skate athletes participated in the longitudinal study, which assessed the gut microbiome and physical fitness such as aerobic capacity and anaerobic power in the general and specific preparation phase of training periodization. The gut microbiome was analyzed using 16S rRNA sequencing.

**Results:**

The cross-sectional study revealed significant differences in *Prevotella, Bifidobacterium, Parabacteroides,* and *Alistipes* genera and in enterotype distribution between transition and preparation season phase periodization. In the longitudinal study, training phase periodization altered the level of *Bacteroides*, *Blautia*, and *Bifidobacterium* in the microbiome. Such changes in the microbiome were significantly correlated with alternations in aerobic capacity and tended to correlate with the anaerobic power.

**Discussion:**

These findings suggest that periodization alters the gut microbiome abundance related to energy metabolism and trainability of physical fitness. Athlete's condition may thus be mediated to some extent by the microbiota in the intestinal environment.

## 1. Introduction

The gut microbiota lives in the human gastrointestinal tract; they decompose dietary fiber that the host cannot digest. Further, they are involved in the production of short-chain fatty acids and vitamins that are beneficial to the host. Intestinal bacteria are increasingly reported to be responsible for regulating other biological functions such as immune functions, enzyme activity, and hormone secretion (1). The gut microbiome forms a complex ecosystem and the composition, profile, and diversity of gut bacteria as an aggregate reflects the host health status (2), i.e., loss of diversity in the gut microbiome and subsequent imbalance can give rise to immune allergies and metabolic disorders leading to obesity, metabolic syndrome, and lifestyle-related diseases (3–5). Contrarily, the gut microbiome also plays adaptation in the compositional profile owing to aging, diet habit, or drug metabolism (6, 7). Exercise has been suggested to alter in the composition and function of the gut microbiome, along with promoting energy metabolism and improving physical function (8). A report by Estaki et al. indicates that the gut microbiome profile in healthy individuals is associated with maximal oxygen uptake (VO_2_max), which is an index of aerobic fitness (9). However, there are few reports regarding the gut microbiome in elite athletes; therefore, elucidation of the gut microbiome composition in athletes remains a topic of interest (8).

Athletes require high-intensity training to improve their physical performance. However, the physical burden of high-intensity training can worsen the physical conditions of athletes (10). Condition deterioration is caused by systemic dysfunction, including the cardiovascular and digestive systems, endocrine responses, and autonomic nervous activity (11), which may manifest as decreased fitness, diminished immune function, increased inflammatory responses, and gastrointestinal disorders, among others (12). In other words, athletes who are in poor condition often complain of decreased heart rate (HR) during exercise; they may also present gastrointestinal symptoms such as diarrhea or stomach aches (10, 13). Further, 30%–50% of athletes are reported to suffer from gastrointestinal symptoms during their training periods (14). Taken together, it seems important to examine the possible relationships between physical fitness and the intestinal environment. The gut microbiome in elite rugby athletes is reported to demonstrate high diversity and is associated with upregulation of pathways responsible for amino acid biosynthesis, carbohydrate metabolism, and short-chain fatty acid synthesis (15). Another study has reported that the extent of training by college swimmer athletes exhibited a positive correlation with the gut microbiome involved in short-chain fatty acid synthesis (16). Training volume and intensity vary from extensive to intensive workload or general to special tasks corresponding to their sports' seasonal periodization structured into macro- (a few months) and meso- (a few weeks) cycles for best performance and attenuating overtraining (17). Taken together, the gut microbiome of athletes may be related to changes in physical fitness and/or condition. However, the changes occurring in the gut microbiome of athletes with changes in fitness during training periodization remain to be studied.

Therefore, the purpose of this study was to investigate the effect of training periodization in athletes on the gut microbiome along with physical fitness. Fecal collection and physical fitness measurements were conducted during separate periodization in elite international level athletes using cross-sectional and longitudinal study designs. We especially focused on the phases of training for elite short-track ice skaters, who require both aerobic and anaerobic capacity because they perform in races involving short, middle, and long-distance skating. We then examined the interrelationship among gut microbiome compositional profiles, stool conditions, and aerobic and anaerobic fitness.

## 2. Materials and methods

### 2.1. Participants

The present study used a cross-sectional and a longitudinal design. We recruited Japanese elite athletes who were at the international level and belonged to the national team in their respective sport. In total, 84 athletes (aged 24 ± 5 years, 49 men and 35 women) across different sports participated in the cross-sectional study and 10 elite short-track speed skaters (aged 22 ± 4 years, 4 men and 6 women) participated in the longitudinal study. This study was performed in accordance with the Declaration of Helsinki and was approved by the ethic committee of the Japan Institute of Sports Sciences (049-01 and 049-02) and National Institute of Biomedical Innovation, Health and Nutrition (KENEI 91). All athletes provided written informed consent prior to participating in the study.

### 2.2. Procedures

#### 2.2.1. Cross-sectional study

Eighty-four athletes were separated into a macro-cycle of training periodization including a transition period (*n* = 22) (10 men and 12 women) and preparation period (*n* = 62) (25 men and 37 women). The participants visited the laboratory center once during their training periodization, and fecal samples were collected. The participants belonged to the national team as Olympic reinforcement-designed athletes in one of following sports; Alpine snowboarding, fencing, jumping events, racewalking, rhythmic gymnastics, short distance running events, sailing, short-track speed skating, and soccer.

#### 2.2.2. Longitudinal study

Ten short-track speed skaters (4 men and 6 women) in the preparation period of training periodization visited the laboratory center on two separate meso-cycles at a 3-months interval for general preparation (the beginning of the phase at high volume and low technique training after the transition period) and specific preparation (the end of the phase at low volume and high technique training before the competition period) phase. Before and after the longitudinal periodization, the participants were assessed for physical fitness over two days, an aerobic maximal exercise test on the first day and anaerobic supramaximal pedaling for 90 s on the second day. Further, the fecal samples were collected within 5 days of physical fitness evaluation in each meso-cycle phase of periodization.

### 2.3. Measurement

#### 2.3.1. Gut microbiome

Fecal samples were collected in commercial vials containing 3 ml guanidine thiocyanate (GuSCN) solution (TechnoSuruga Laboratory Co., Ltd., Shizuoka, Japan), mixed by vortexing, and stored at room temperature (25 °C). The gut microbiome was analyzed as described previously (18). Briefly, the mixture was homogenized using the bead beating method on a Cell Destroyer PS1000 (Bio Medical Sciences, Tokyo, Japan) and DNA was extracted using a Gene Prep Star PI-80X device (Kurabo Industries LTD.). Samples were stored at −30 °C until use. The V3-V4 region of the 16S rRNA gene was amplified from fecal DNA samples using the following primers: forward: 5′-TCGTCGGCAGCGTCAGATGTGTATAAGCGACAGCCTACGGGNGGCWGCAG-3′, and reverse: 5′-GTCTCGTGGGCTCGGAGATGTGTATAAGAGACAGGACTACHVGGGTATC TAATCC-3′. The cycling parameters were as follows: initial denaturation at 95 °C for 3 min, followed by 25 cycles of denaturation (95 °C for 30 s), annealing (55 °C for 30 s), and extension (68 °C for 1 min), and a final extension step at 68 °C for 5 min. After adding the sequencing adapters, the amplicons were sequenced using the Illumina MiSeq platform (Illumina Inc., San Diego, USA) according to the manufacturer's instruction. Sequence reads from Illumina MiSeq were analyzed using Quantitative Insights Into Microbial Ecology (QIIME) and QIIME Analysis Automating Script (Auto-q) as previously described (19). The obtained paired-end reads were selected, and chimeric sequences were removed using USEARCH v6.1. Open-reference operational taxonomic unit (OTU) picking, and taxonomy classification were performed based on sequence similarity (>97%) by using UCLUST software with the SILVA v128 reference sequence.

#### 2.3.2. Stool condition and diet questionnaire

We evaluated the stool frequency, volume, color, and form using a previously developed assessment tool and validated against objective measurements of stool characteristics including stool weight, moisture, hardness, and color in adults (20). The frequency of excretion was evaluated as number of excretions per week using six responses ranging from less than 2 times to more than 7 times. Stool volume was estimated by number of fecal units (response number 1–8, from 1, 0.5 units to 8, >4 units) based on the model stool unit (2 cm diameter × 10 cm length, cylindrical in shape). Stool form was measured based on the modified Bristol stool form scale (21). The seven types range from very hard (type 1) to very loose (type 7). For stool color assessment, the closest of the six colors indicated on the stool assessment tool compared to the actual color was selected according to the color standard Z8721: 1, 5Y8/12 (yellow); 2, 2.5Y7/12 (light yellowish-brown); 3, 10YR5/8 (yellowish-brown); 4, 7.5YR7/12 (brown); 5, 5Y4/4 (greenish-dark brown); 6, 2.5GY4/3 (dark brown). Furthermore, stool odor was evaluated on the scale from 1 to 3 (1, odorless; 2, normal; 3, stronger than usual). Habitual diet during the preceding month were assessed by the brief self-administered diet history questionnaire (BDHQ) (22). The daily intake of protein, fat, and carbohydrate were calculated from the record.

#### 2.3.3. Physical fitness

In the longitudinal study, before and after training periodization, the participants were measured for body composition and for aerobic and anaerobic fitness. Body composition was assessed using air displacement plethysmography (BODPOD; Cosmed, Concord, USA), and percentage body fat and fat-free mass was calculated. Aerobic fitness was measured by a maximal graded exercise test using a cycle ergometer to determine the VO_2_max. After sufficient warm-up, the exercise started at 120 W for male and 80 W for female athletes, and the work load was increased by 40 W every 3 min until stage 7 and by 20 W every 3 min above stage 8 until they reached an exhaustive state; if one of following criteria: unable to maintain cadence (<80 rpm) for several seconds, attainment of age-predicted maximal HR (220 - age), respiratory exchange ratio >1.1, rating of perceived exertion >19, or clinical complaints of exhaustion by the participants. HR was monitored continuously with wireless Polar sensor (RS800CX; Polar Electro, Finland). The participants were instructed to maintain a cadence of 80 rpm during the test. We measured the average oxygen uptake every 30 s during the exercise test using an online computer-assisted circuit spirometer (AE300S; Minato Medical Science, Osaka, Japan) and evaluated the highest value as VO_2_max. Ventilatory threshold (VT) was calculated using a regression analysis of the slopes of carbon dioxide production, oxygen ultake, and the minute ventilation plot. In addition, we measured blood lactate concentration at the end of each stage and calculated HR at onset blood lactate accumulation (OBLA: defined as 4 mmol/L) as an index of submaximal aerobic fitness. Anaerobic fitness was measured by a 90 s supramaximal pedaling test on a cycle ergometer (Power Max III; Konami LTD., Tokyo, Japan). The load was set at 7.5% kp of their body weight. The participants were strongly encouraged to maintain pedaling at a maximal effort throughout the entire exercise from the beginning to end. We evaluated the average power output for the initial 30 s.

### 2.4. Statistical analyses

All statistical analyses were performed using SPSS version 24 (IBM Inc., Chicago, USA) and R version 3.4.2. In the cross-sectional study, continuous, ordinal, and categorical variables were analyzed using an unpaired Student *t*-test, Mann–Whitney test, and *χ*^2^ test. In the longitudinal study, we used a paired Student's *t*-test or Wilcoxson rank test and r or Cramer's effect size (ES) was calculated to identify the changes in variables before and after training periodization. Univariable Spearman correlation analyses were used to determine the relation between variables of interest. For analyzing the changes in stool form (Bristol scale), direction to the ideal normal form (Type 4) was regarded as the degree of improvement. In the longitudinal study, one athlete dropped out because of lower limb injury (*n* = 9), one athlete canceled the measurement of anaerobic power pedaling owing to lower limb pain, and another athlete lost the response sheet of the stool condition questionnaire report; the data of anaerobic power and stool condition were analyzed for 8 participants (*n* = 8). Statistical analyses of the gut microbiome were performed using R software. Principal coordinate analysis (PCoA) based on the Bray-Curtis distance metric was performed using multivariate techniques of global view using the R packages vegan and ade4. Jensen–Shannon divergence was used for enterotype analysis as previously described (23). The top 20 most abundant genera in the microbiome were presented in boxplot analyses. Data are expressed as the means ± SD and number of counts (distribution %) as appropriate. Statistical significance was set *a priori* at *P* < 0.05 for all comparisons.

## 3. Results

**Figure 1:**
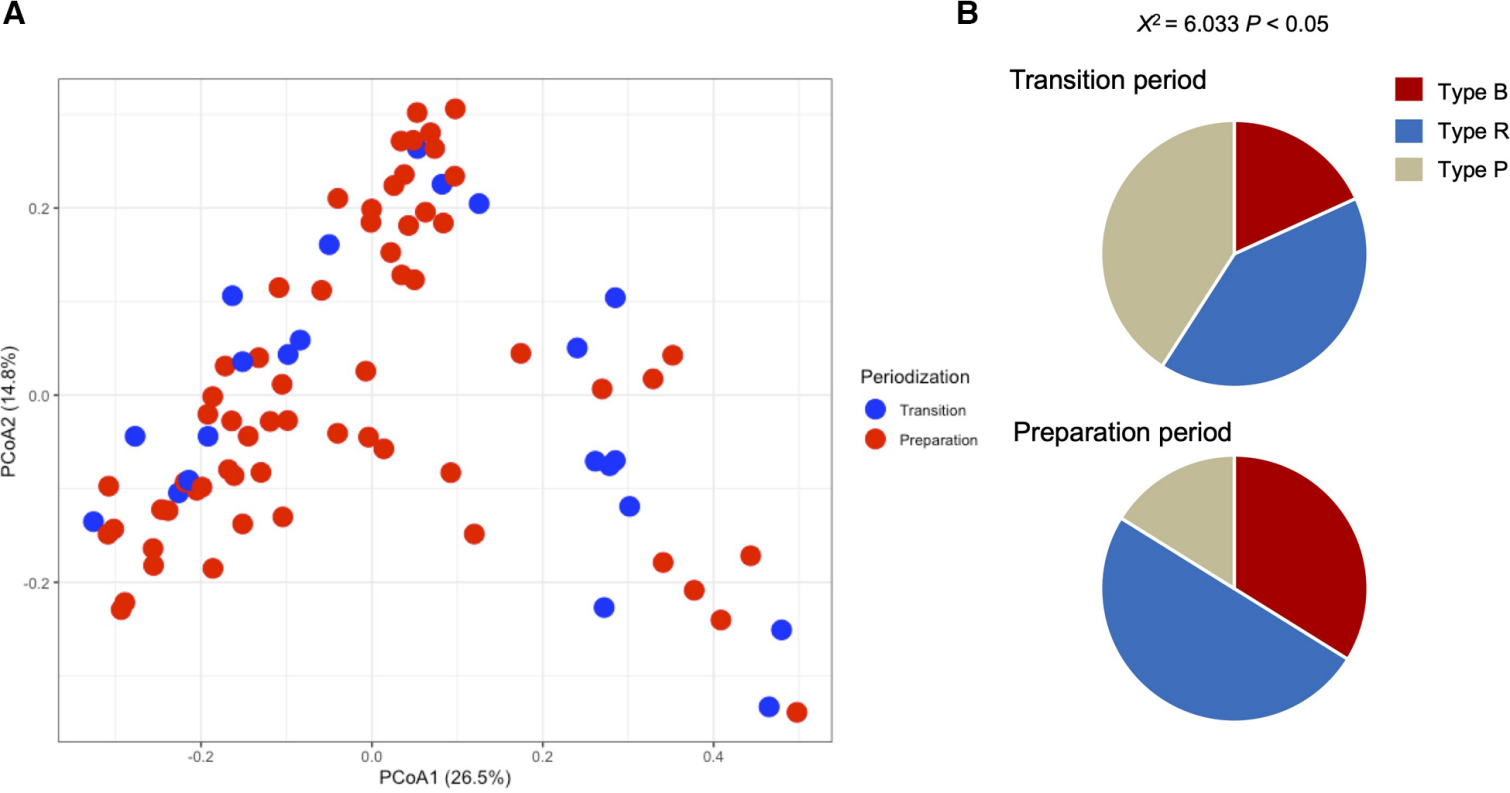
Gut microbiota profile presented by principal coordinate analysis (PCoA, genus level) (**A**) and the frequency of each enterotype, *Bacteroides* (Type B), *Ruminococcus* (Type R), and *Prevotella* (Type P), in the transition and preparation period (**B**).

**Figure 2:**
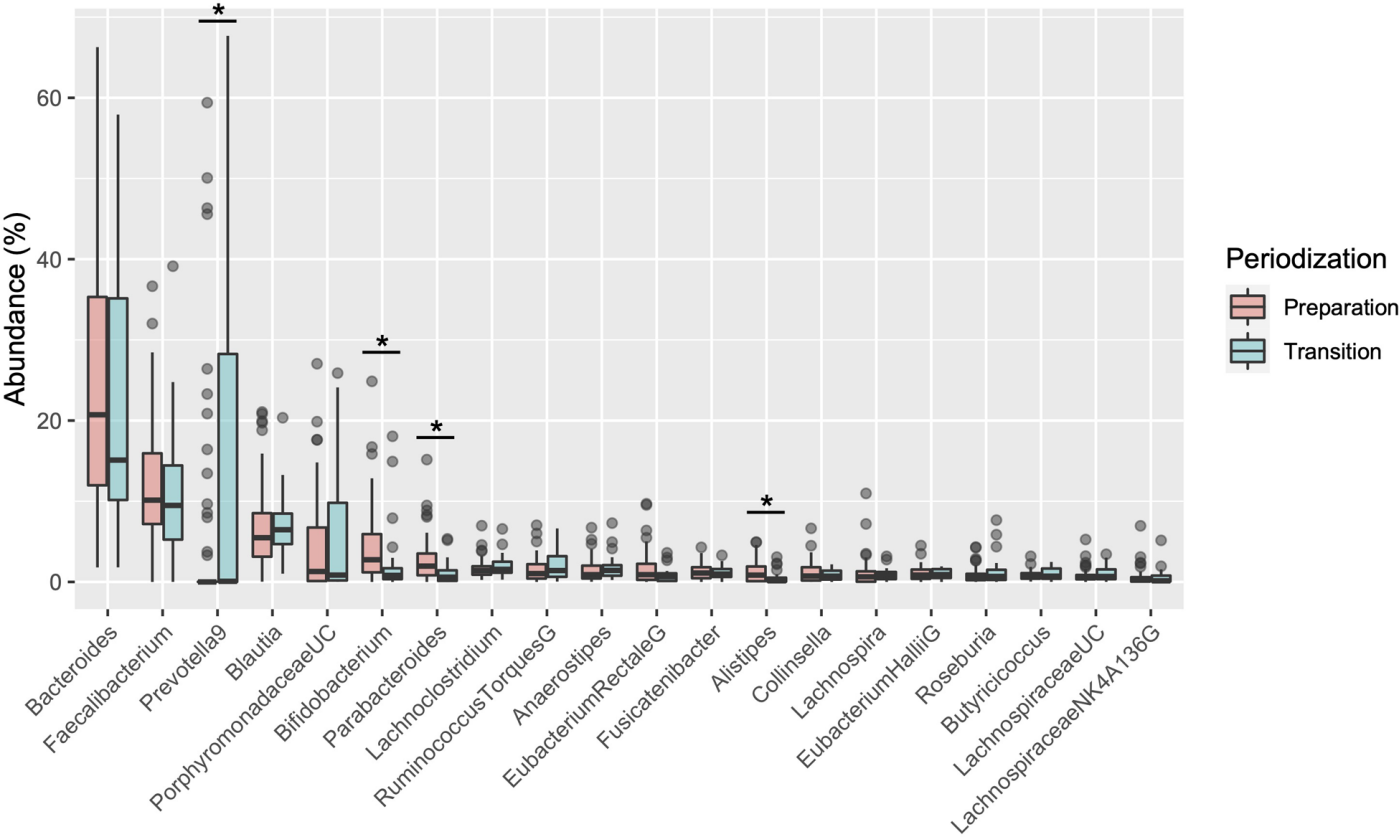
Boxplot of differences in the gut microbiota relative abundance at the genus level. *indicates a significant difference (*P* < 0.05) between transition and preparation periodization.

Table 1 shows the participant characteristics between the transition and preparation period in the cross-sectional study. The percentage of female, daily intake of diet and macronutrients, and stool conditions were not different between the two groups. There were no differences in the gut microbiome profile between sexes. Figure 1 depicts the gut microbiome community structure by genus level PCoA and enterotype analysis clustering using Jensen–Shannon divergence. The enterotype was characterized by the predominance of three genera, *Bacteroides* (B), *Prevotella* (P), and *Ruminococcus* (R). Athletes in their preparation period exhibited a higher frequency of type B and a lower frequency of type *P* (*X*^2^ = 6.033, *P* < 0.05) (Figure 1B). Figure 2 shows the comparison of the most abundant gut microbiome at the genus level in the cross-sectional macro-cycle periodization in the transition and preparation period. The distribution of *Prevotella* was significantly lower and that of *Bifidobacterium*, *Parabacteroides*, *Alistipes* was significantly higher in the preparation period than in the transition period (Figure 2).

**Table 1:** The participants’ characteristics and stool condition between transition and preparation period of training periodization.

	Transition period		Preparation period	
Number, *n*	22		62	
Women, *n* (%)	12	(55)	37	(60)
Height, cm	168	(6)	171	(4)
Weight, kg	64	(7)	65	(9)
Macronutrients				
Energy, kcal	2,444	(1,045)	2,351	(899)
Carbohydrate, %	56	(8)	58	(8)
Protein, %	17	(4)	16	(3)
Fat, %	26	(6)	26	(6)
Saturated fat, *g*	19.3	(10.0)	18.1	(7.6)
Monosaturated fat, *g*	24.5	(12.0)	24.7	(10.1)
Polysaturated fat, *g*	16.9	(8.8)	16.2	(6.1)
Cholesterol, *g*	640	(408)	515	(228)
Fiber, *g*	17.2	(8.4)	14.3	(6.7)
Stool conditions				
Frequency, times/week	6.5	(1.0)	6.3	(1.3)
Volume, units	4.0	(2)	3.9	(1.2)
Form, units	4.1	(1.1)	3.9	(1.2)
Color, units	4.0	(0.9)	4.1	(1.0)
Odor, *n* (%)				
Strong	6	(27)	11	(18)
Normal	15	(68)	49	(79)
Odorless	1	(5)	2	(3)

Data are presented as the means (SD), or number of counts (distribution %), as appropriate.

**Figure 3:**
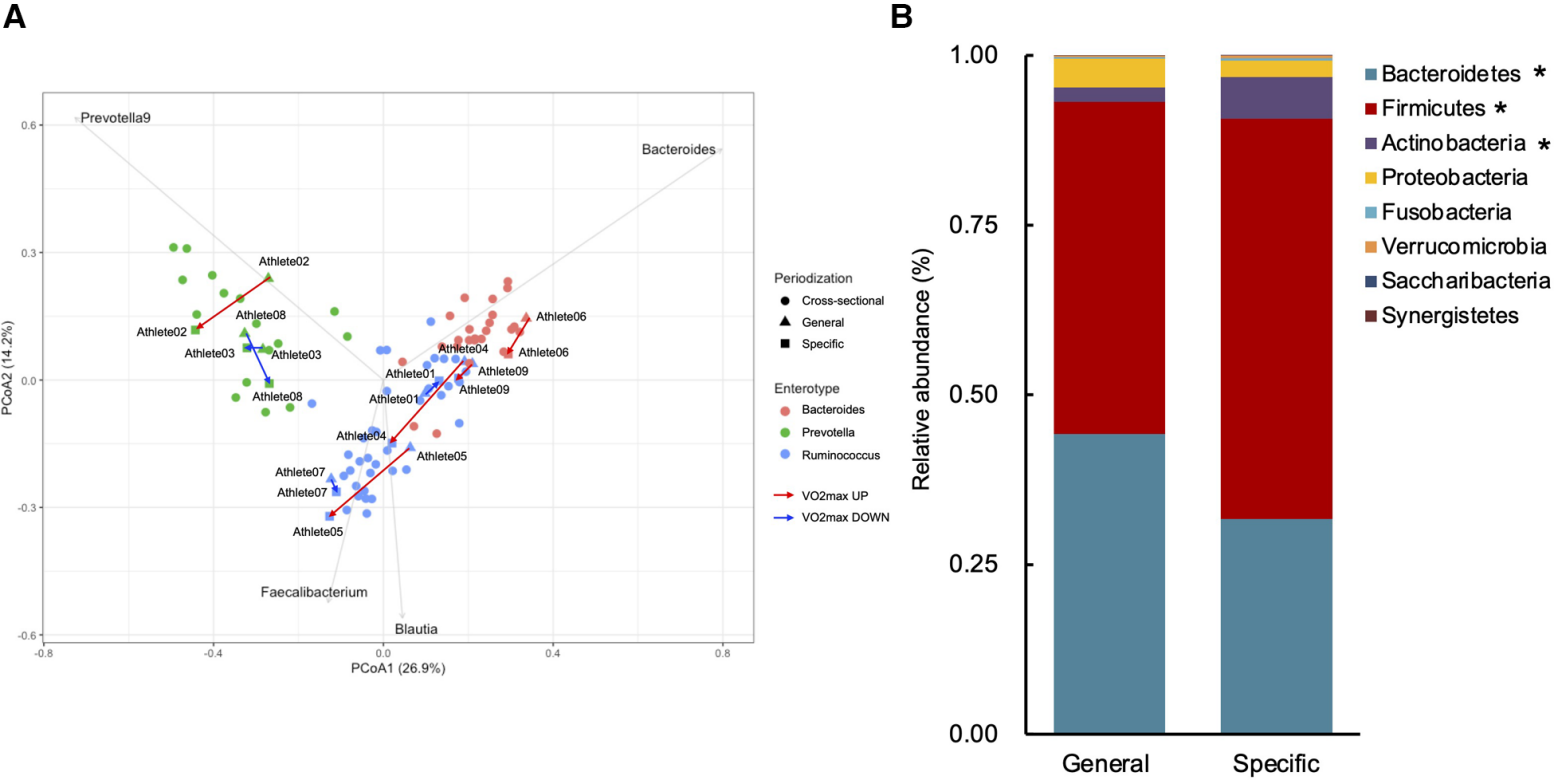
Gut microbiota profile presented by principal coordinate analysis (PCoA, genus level) (**A**). Triangles indicate the preparation training phase, squares indicate the transition training phase, and circles indicate the cross-sectional study. Gray arrows indicate enterotype drivers; *Bacteroides* (pink), *Prevotella* (green), and *Rumicnococcus* (*Blutia & Faecalibacterium*) (blue). Red arrows indicate increased VO_2_max athletes and blue arrows indicate decreased VO_2_max athletes. Comparison of gut microbiota relative abundance at the phylum level (**B**). *indicates a significant change (*P* < 0.05) between the general and specific preparation phase periodization.

**Figure 4:**
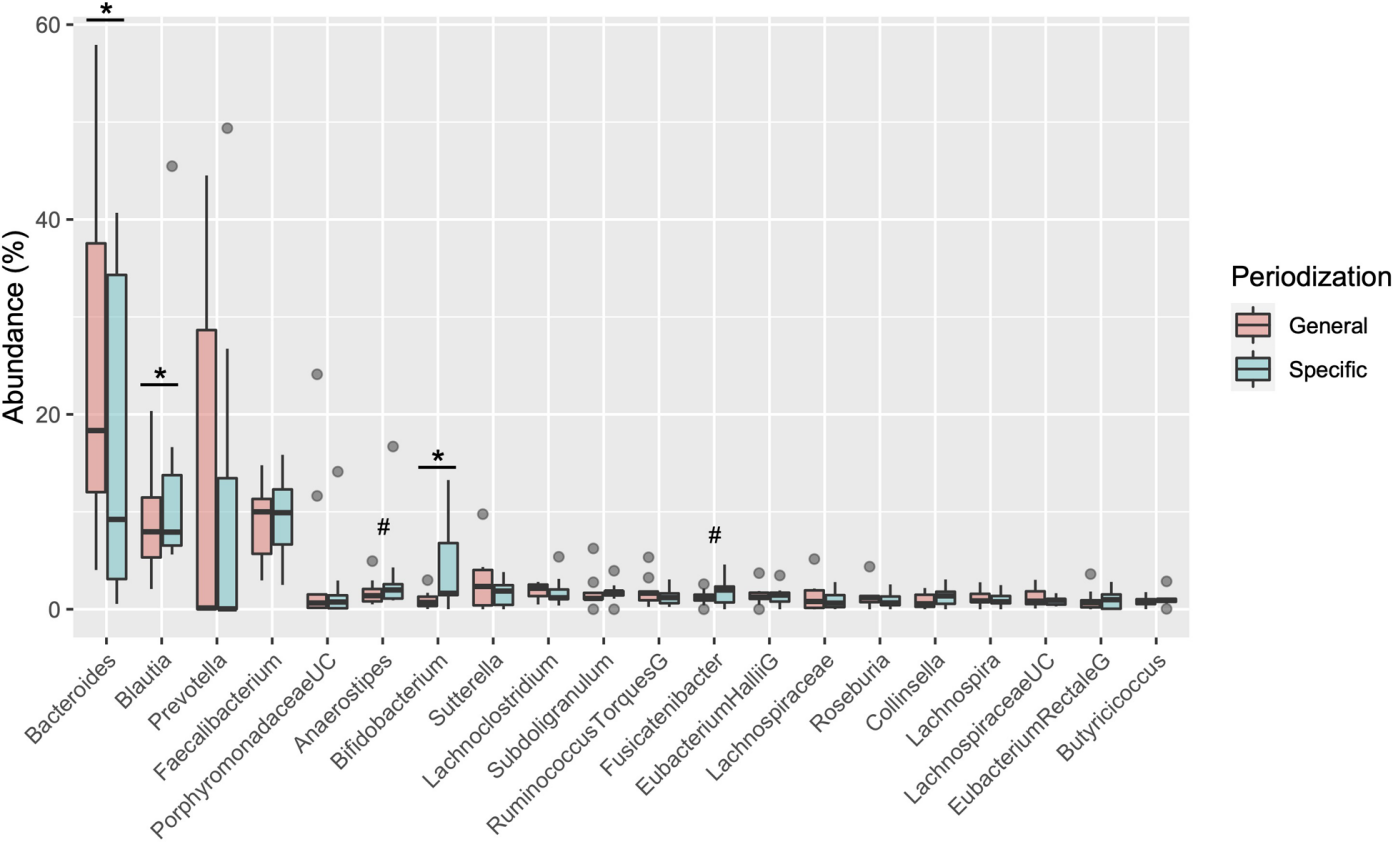
Boxplot of changes in gut microbiota relative abundance at the genus level. *indicates a significant difference (*P* < 0.05) and # indicates tended to a difference (*P* = 0.066) before and after the periodization.

**Figure 5:**
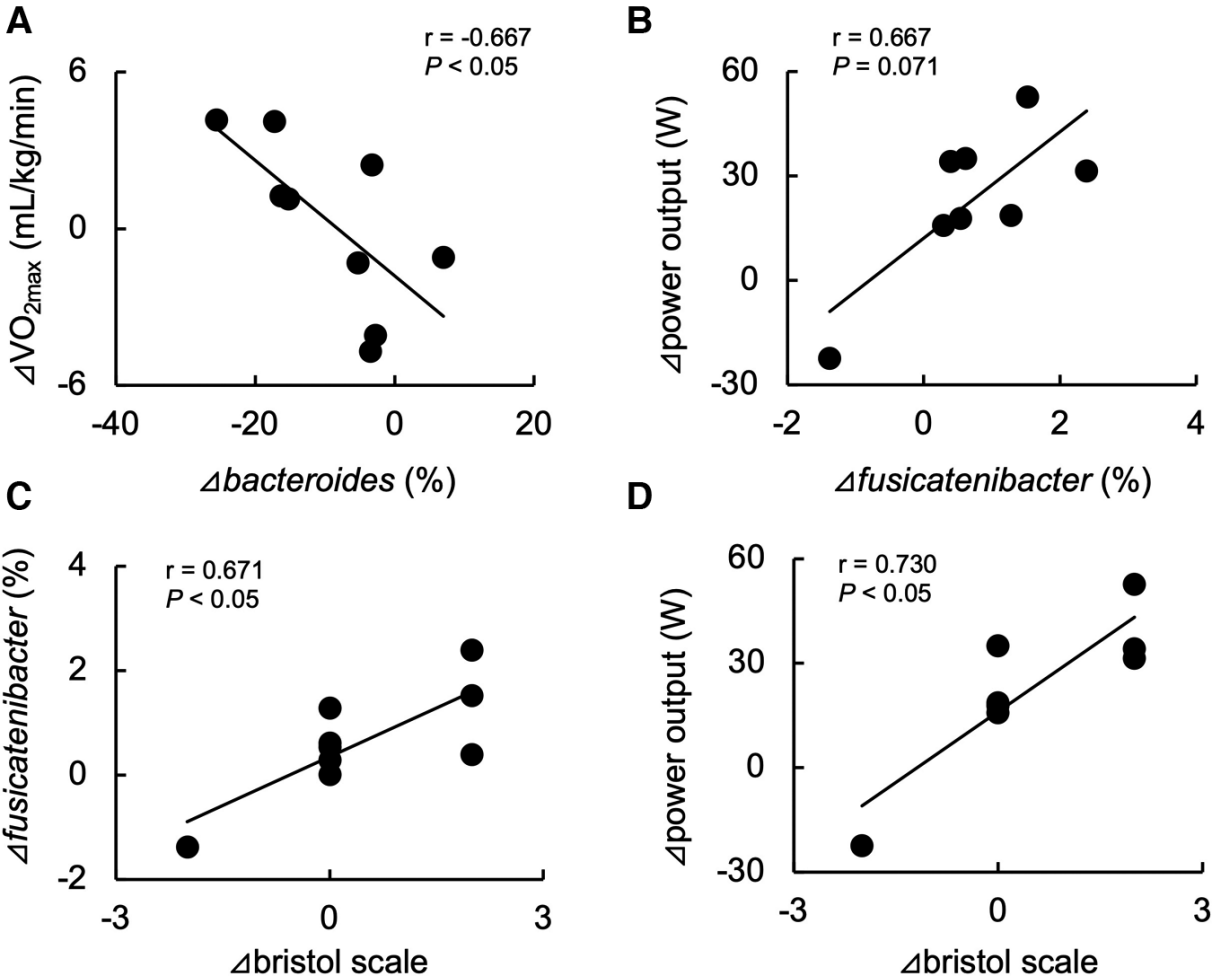
Relationship between changes in maximal oxygen uptake (VO_2_max) and Bacteroides abundance (**A**), anaerobic power output and Fusicatenibacter abundance (**B**), and improvements in the Bristol scale and Fusicatenibacter abundance (**C**) and anaerobic power output (**D**) before and after the preparation phase periodization.

Table 2 shows the changes in the participant characteristics in the longitudinal study. The percentage of body fat was significantly lower (*P* = 0.012, ES = 0.70), and fat-free mass was significantly higher (*P* = 0.006, ES = 0.75) in specific preparation phase than that of general preparation phase, however, body weight was not changed (*P* = 0.162, ES = 0.35). In the transition training phase, submaximal aerobic capacity of HR at 4 mmol/L of lactate and anaerobic power output was significantly increased (*P* = 0.006, ES = 0.77 and *P* = 0.011, ES = 0.74, respectively), VT tended to increase (*P* = 0.055, ES = 0.54), but VO_2_max did not change significantly (*P* = 0.425, ES = 0.07). There were no significant changes in diet macronutrients and stool conditions such as the frequency (*P* = 0.257, ES = 0.38), volume (*P* = 0.395, ES = 0.28), color (*P* = 0.785, ES = 0.09), form (*P* = 0.317, ES = 0.33), and odor (*P* = 0.624, ES = 0.32) (Table 2). Figure 3 depicts the gut microbiome composition profile in each athlete. PCoA of microbial genus abundance indicates that the microbiota profile composition was changed in athlete in the longitudinal study besides over the athletes in the cross-sectional study (Figure 3A). All athletes in the longitudinal study exhibited that the changes were within same enterotype cluster. Moreover, athletes with increased VO_2_max showed changes in the adverse direction for the genus *Bacteroides* (negatively both PCoA1 and PCoA2 component). Figure 3B shows the longitudinal changes in the gut microbiome at the phylum level during the general and specific preparation phase. *Bacteroidetes* phylum distributions were significantly decreased (*P* = 0.028, ES = 0.73), whereas *Firmicutes* and *Actinobacteria* phylum distributions were significantly increased (*P* = 0.028, ES = 0.73 and *P* = 0.021, ES = 0.77, respectively) in the specific phase compared to those in the general phase. Moreover, at the genus level, the distribution of *Bacteroides* was significantly decreased (*P* = 0.028, ES = 0.69), whereas that of *Blautia* and *Bifidobacterium* was significantly increased (*P* = 0.015, ES = 0.81 and *P* = 0.021, ES = 0.80, respectively) after training periodization (Figure 4). A tendency of increase in *Fusicatenibater* and *Anaerostipes* distribution was observed (*P* = 0.066, ES = 0.61 and *P* = 0.066, ES = 0.61, respectively). The relationships between the gut microbiome and physical fitness, in which changes were examined before and after preparation periodization, are shown in Figure 5. Significant correlations were evidenced between the changes in VO_2_max and *Bacteroides* (*r* = −0.667, *P* < 0.05) (Figure 5A), and a tendency of correlation was observed in the changes in anaerobic power output and *Fusicatenibacter* (*r* = 0.677, *P* = 0.071) (Figure 5B). Improvement in the Bristol scale was significantly correlated with changes in *Fusicatenibacter* abundance (*r* = 0.671, *P* < 0.05) (Figure 5C), and anaerobic power output (r = 0.730, *P* < 0.05) (Figure 5D).

**Table 2:** The participants’ characteristics and stool conditions in general and specific preparation phase of training periodization.

	General preparation phase		Specific preparation phase	
Women, *n* (%)	5	(56)		
Height, cm	164	(5)		
Weight, kg	59	(6)	60	(5)
Fat, %	13.3	(4.5)	11.4	(5.0)*
Fat-free mass, kg	51.6	(6.9)	53.2	(6.3)*
HR at OBLA, bpm	167	(9)	171	(11)*
VT, ml/kg/min	44.2	(7.0)	45.6	(6.2)
VO2max, ml/kg/min	57.6	(7.9)	57.8	(6.8)
Maximal pedaling anaerobic power^a^, W	579	(119)	602	(110)*
Macronutrients				
Energy, kcal	2,682	(985)	2,662	(735)
Carbohydrate, %	56	(8)	59	(7)
Protein, %	19	(5)	17	(4)
Fat, %	25	(4)	24	(4)
Carbohydrate, %	56	(8)	59	(7)
Saturated fat, *g*	21.2	(11.1)	20.1	(7.5)
Monosaturated fat, *g*	25.7	(12.3)	24.8	(9.8)
Polysaturated fat, *g*	18.1	(11.2)	17.2	(6.8)
Cholesterol, *g*	686	(431)	635	(275)
Fiber, *g*	21.0	(8.2)	19.6	(7.0)
Stool condition				
Frequency, times/week	6.5	(0.8)	6.1	(1.5)
Volume, units	3.5	(2.1)	4.0	(2.1)
Color, units	4.5	(0.8)	3.6	(0.9)
Form, units	4.6	(1.2)	4.1	(0.8)
Odor, *n* (%)				
Strong	3	(38)	3	(38)
Normal	5	(63)	4	(50)
Odorless	0	(0)	1	(13)

Data are presented as the means (SD), or number of counts (distribution %), as appropriate.

HR, heart rate; OBLA, onset of blood lactate accumulation; VT, ventilatory threshold.

^a^ *n* = 8.

* *P* < 0.05 vs. preparation phase.

## 4. Discussion

The present study investigated the impact of training periodization on the gut microbiome, stool condition, and physical fitness in elite Japanese athletes. The salient findings of the study were as follows: we observed significant differences in enterotype distribution and the abundance of genera such as *Prevotella*, *Bifidobacterium*, *Alistipes*, and *Parabacteroides* in the microbiome during the macro cycle of periodization in the cross-sectional design. We also observed changes in *Bacteroides*, *Blautia*, and *Bifidobacterium* in the microbiome along with a tendency for increase in the genera *Fusicatenibacter* and *Anaerostipes* in the longitudinal phase of the preparation period. In addition, a decrease in the genus *Bacteroides* was correlated with a change in VO_2_max, and an increase in *Fusicatenibacter* tended to be associated with enhancement of anaerobic power. These results suggest that changes in the gut microbiome of athletes by season and/or training may be associated with their physical fitness and condition.

Emerging evidence has demonstrated the relation among the gut microbiome, lifestyle, and several diseases; researchers have also focused on physical fitness and exercise training (8). In the present study, a difference was observed in the prevalence of gut microbial clusters categorized as *Bacteroides*, *Prevotella*, *Ruminococcus* enterotypes (4). We observed a higher percentage of the *Bacteroides* enterotype and a lower percentage of the *Prevotella* enterotype in the preparation period than that in the transition period of the cross-sectional design. The *Bacteroides* enterotype is associated with long-term consumption of a protein- and animal fat-rich diet, whereas the *Prevotella* enterotype is associated with a carbohydrate- and fiber-rich diet (24). *Prevotella* species are reported to exert a beneficial effect in protecting against *Bacteroides*-induced glucose intolerance and improving glucose metabolism (25). Based on the genus composition of the gut microbiome, abundance of *Prevotella* was significantly higher in the transition period than in the preparation period (Figure 2). A previous study showed that diet intervention contained high-fat/low-fiber or low-fat/high-fiber for 10 days affected gut microbiome composition but could not change the enterotype in healthy adults (24). In the current study, we first performed the cross-sectional and longitudinal changes in the athlete's microbiome without dietary intake variance. Interestingly, the results revealed that the changes in the microbiome components depend on individual variability and that some skate athletes showed large changes inter-individually, but no changes were found in the enterotype. Taken together, these results indicate that the gut microbiome profile show changes as large as inter-individual differences, but these changes remain within the same cluster.

The abundances of *Bifidobacterium*, *Prevotella*, *Parabacteroides*, and *Alistipes* genera were significantly different in the cross-sectional study between the transition and preparation period; in the longitudinal design, the abundance of *Bifidobacterium* and *Blautia* increased and that of the *Bacteroides* genus decreased after the specific preparation phase. *Bifidobacterium* was detected as a significant genus in both the cross-sectional and longitudinal studies. *Bifidobacterium*, *Prevotella* or *Blautia* are the predominant bacteria often found in the intestine of Japanese individuals, and are considered to have beneficial effects on intestinal homeostasis through enhanced energy metabolism, anti-inflammatory action, and maintenance of intestinal pH, by fermenting polysaccharides into organic acids such as lactic acid, acetic acid, and butyric acid (11, 26). The genus *Bifidobacterium* has been previously used as a probiotic, and dietary supplements combined with the genera *Lactobacillus* and *Streptococcus* have been reported to extend athlete exhaustion time under heat stress in long-distance running (27). These findings suggest that training for preparation periodization induces changes in the specific microbiome composition including *Bifidobacterium,* rather than in a single genus, which may have an ergogenic effect on physical function.

Cronin et al. reported that no significant changes in gut microbiome were observed in healthy individuals even after combined aerobic and resistance exercise at mild intensity 3 day/week for eight weeks (28). Meanwhile, Allen et al. examined the compositional profile of the gut microbiome in lean and obese people and reported that the gut microbiome composition was significantly different before the beginning of exercise training; however, there was no difference after six weeks of moderate-intensity aerobic exercise training, and the abundance of *Bacteroides* and *Faecalibacterium* was changed (29). Karl et al. showed that high-intensity interval training in military personnel altered the abundance of *Bacteroidetes* and *Firmicutes* phyla over a short period of four days, significantly changing the compositional profile of the gut microbiome (30). In a study on rower athletes, the gut microbiome exhibited a decreasing tendency in the abundance of *Bacteroides,* and an increasing tendency in abundance of *Rosebuila* and *Subdoligranulim* in all athletes during the competition season with improved physical condition (31). In the present longitudinal study, we demonstrated decreased an abundance of the phylum *Bacteroidetes* and genus *Bacteroides,* and an increased abundance of the phylum *Firmicutes* in the specific preparation phase, which was consistent with previous studies. Similarly, the increased *Firmicutes* and *Bacteroidetes* ratio (F/B) has been associated with higher VO_2_max in general heathy young adults (32). On the contrary, the compositional ratio of F/B is elevated in obese populations with a body mass index (BMI) >30 (33, 34). However, in Japanese cohorts, as obese individuals forma minority, no relationship between F/B and BMI has been demonstrated in the normal range of BMI (35). As the consensus of this association could not be confirmed, the increased F/B in the longitudinal study could be interpreted as a gut microbiome adaptation contributing to the efficient absorption of ingested micronutrients with much energy expenditure during preparation periodization.

A previous study has reported that the aerobic fitness of VO_2_max is positively correlated with the microbiome, and is related to the production of short-chain fatty acids, which serve as energy sources through the genera *Lacnospiraceae*, *Roseburia*, and *Clostridiales* (9). In the current study, although VO_2_max was not significantly altered by training periodization, the changes in individual VO_2_max were negatively correlated with the abundance of *Bacteroides*. Furthermore, our PCoA supported this relationship, our findings indicated that increased VO_2_max showed the same change direction of decreased in both PCoA1 and PCoA2 components which were mainly against the predominance of *Bacteroides* (Figure 3). *Bacteroides* is known to play a role in the metabolism of organic acids, bile acids, and proteins, leading to regulation of energy homeostasis in the host (36). An overabundance of protein promotes fermentation of amino acids by the genus *Bacteroides* in the gastrointestinal tract, which then accumulates toxic by-products such as amines, phenols, hydrogen sulfide, indoles, and ammonia (37). An excessive increase in circulating ammonia during intensive exercise has been associated with central fatigue and with reduced exhaustion performance (38). Taken together, a decrease in the genus *Bacteroides* seems to attenuate ammonia elevation during exercise and changes the maximal graded exercise test performance. On the other hand, there is no correlations between submaximal aerobic capacity such as VT or OBLA and microbial changes. These results suggest that changes in the abundance of *Bacteroides* among other gut microbiome components are partly related to the trainability of maximal rather than submaximal aerobic fitness in athletes.

Anaerobic power performance as determined by supramaximal pedaling was significantly increased after the specific preparation phase, and the degree of change tended to be related to elevated levels of the genus *Fusicatenibacter*, which showed an increasing tendency among gut microbiomes. Bacteria of the genus *Fusicatenibacter* promote lactose decomposition and produce organic acids in the intestine, including lactic acid and short-chain fatty acids (39). These organic acids, when absorbed by the large intestine, pass through the hepatic portal vein, and are metabolized to glycogen and fatty acids in the liver and muscles to provide energy (40). Gut bacteria may thus be associated with glycolytic energy metabolism mechanisms such as glycogen storage and lactic acid metabolism in muscles.

In this study, we used the Bristol scale (41) to evaluate the stool form condition status. The Bristol scale assesses the form of fecal matter across seven types, ranging from hard stools with long intestinal transit time and high water absorption to watery stools with short intestinal transit time and low water absorption (42). A study in Japanese adults showed that stool form is related to the intestinal environment and that quality-of-life indices are rated lower for individuals with constipation (43). In this study, no changes were observed in the mean values of the Bristol scale scores during the periodization. However, analysis of the degree of improvement in the Bristol scale with athlete periodization revealed a significant correlation with changes in the genus *Fusicatenibacter* and anaerobic power performance when change from hard or watery stools to normal fecal form in the ideal direction was determined as the degree of improvement. However, this relation was not observed in aerobic fitness. The genus *Fusicatenibacter* may mediate the link between improvement of stool condition and enhancement of anaerobic power performance. Taken together, stool form may be a surrogate marker for evaluating gastrointestinal and physical conditions.

The present study had some limitations. First, this study was conducted small sample size. It is reported that sex, sports characteristics, or sports specific diet would affect gut microbial structure (35, 44). Because we recruited only elite athletes who are international level in various sports, the number in each sport was very small, even in longitudinal study. For that reason, we might not have found differences in gut microbiome or macronutrient among sexes and sport events in the present study. Therefore, the results of present study may limit to generalize our findings to specific athlete. Next, this study was observational cross-sectional and longitudinal design and we found aerobic and anaerobic capacity correlated with some gut microbiome or stool condition. Therefore, we cannot establish a causal relationship between physical fitness and stool condition. Furthermore, we did not evaluate mechanistic insight. Barton et al. have reported that gut microbiome of athletes is associated with metabolic pathway including synthesis of organic enzyme, carbohydrate degradation, and short-chain fatty acid compared to sedentary subject (15). Therefore, further studies are warranted to investigate the relationship between physical fitness and gastrointestinal environment with metabolic function in the interventional design.

In conclusion, the gut microbiome of athletes was cross-sectionally and longitudinally examined during training periodization in this study. Certain changes were observed in the gut microbiome including a decrease in abundance of the genus *Bacteroides*, an increase in genus *Blautia* and *Bifidobacterium*, and an increasing trend for the genus *Fusicatenibacter*. Furthermore, the degree of improvement in the altered gut microbiome and defecation status were related to changes in VO_2_max and anaerobic power. These findings indicate that stool condition can be employed to assess the condition of athletes, and suggest the possibility of intervention targeting specific gut microbiome flora for improved conditioning.

## Data Availability

The original contributions presented in the study are included in the article, further inquiries can be directed to the corresponding authors on reasonable request.
